# Cardiotoxic Effects of Antibody Drug Conjugates vs Standard Chemotherapy in *ERBB2*-Positive Advanced Breast Cancer: A Systematic Review and Meta-Analysis

**DOI:** 10.1001/jamanetworkopen.2025.40336

**Published:** 2025-11-09

**Authors:** Lakshya Seth, Aditya Bhave, Sai Kollapaneni, Viraj Shah, Tarek Nahle, Anne Blaes, Susan Dent, Sara A. Hurvitz, Avirup Guha

**Affiliations:** 1 Department of Internal Medicine, University of Texas Southwestern Medical Center, Dallas; 2 Department of Medicine, Division of Cardiology, Medical College of Georgia at Augusta University, Augusta; 3 Department of Medicine, Division of Hematology and Oncology, University of Minnesota, Minneapolis; 4 Wilmot Cancer Center, Department of Medicine, University of Rochester, Rochester, New York; 5 Department of Medicine, UW Medicine, Fred Hutchinson Cancer Center, Seattle, Washington; 6 Cardio-Oncology Program, Department of Medicine, Cardiology Division, Medical College of Georgia at Augusta University, Augusta

## Abstract

**Importance:**

Antibody-drug conjugates (ADCs), such as trastuzumab emtansine and trastuzumab deruxtecan, are effective in treating erb-b2 receptor tyrosine kinase 2 (*ERBB2*)–positive breast cancer (BC) that has progressed on prior *ERBB2*-targeted therapy, warranting evaluation of their cardiotoxic profiles.

**Objective:**

To compare the incidence of cardiotoxic effects of ADCs vs standard-of-care chemotherapy regimens for *ERBB2*-positive locally advanced or metastatic BC.

**Data Sources:**

PubMed, ScienceDirect, Cochrane Library, and ClinicalTrials.gov databases were searched in December 2024 for studies published between 2000 and 2024.

**Study Selection:**

The included studies were (1) phase 3 clinical trials that investigated locally advanced or metastatic *ERBB2*-positive BC; (2) clearly defined left ventricular ejection fraction (LVEF) decrease or heart failure definitions; (3) clearly defined LVEF monitoring frequency by echocardiography or multigated acquisition scan; (4) included studies consisted solely of either trastuzumab emtansine, trastuzumab deruxtecan, or one of the first-line to fourth-line chemotherapy regimens for unresectable stage IV *ERBB2*-positive breast cancer per the 2025 National Comprehensive Cancer Network guidelines; and (5) clearly defined cardiovascular eligibility criteria.

**Data Extraction and Synthesis:**

Data from eligible studies were extracted by 3 reviewers. A random-effects model was used for the pooled analysis. The Preferred Reporting Items for Systematic Reviews and Meta-Analyses (PRISMA) guideline was followed.

**Main Outcomes and Measures:**

The primary outcome was cardiotoxic effects, which were defined as the incidence of LVEF decrease. The pooled analysis was performed using logit-transformed proportions with the inverse variance method and a DerSimonian-Laird random-effects model for between-study variance, with Wilson score 95% CIs.

**Results:**

In this meta-analysis of 9538 patients, a pooled analysis demonstrated a 0.94% (95% CI, 0.56%-1.57%) incidence of LVEF decrease with trastuzumab emtansine, a 4.20% (95% CI, 2.91%-6.01%) incidence with trastuzumab deruxtecan, a 4.85% (95% CI, 3.73%-6.28%) incidence with trastuzumab plus chemotherapy, and a 5.52% (95% CI, 3.41%-8.83%) incidence with trastuzumab plus pertuzumab plus chemotherapy. A trim-and-fill analysis was used if evidence of publication bias was found.

**Conclusions and Relevance:**

This meta-analysis found that trastuzumab emtansine was associated with the lowest incidence of LVEF decrease, and trastuzumab deruxtecan, trastuzumab plus chemotherapy, and trastuzumab plus pertuzumab plus chemotherapy had similar incidences. More research is needed into the cardiotoxic effects of these therapies.

## Key Points

**Question:**

What is the incidence of cardiotoxic effects of novel antibody-drug conjugates compared with the standard-of-care chemotherapy regimens containing trastuzumab for *ERBB2*-positive locally advanced or metastatic breast cancer?

**Findings:**

In this systematic review and meta-analysis of 9538 patients, a pooled analysis demonstrated a 0.94% incidence of left ventricular ejection fraction (LVEF) decrease with trastuzumab emtansine, a 4.20% incidence of LVEF decrease with trastuzumab deruxtecan, a 4.85% incidence of LVEF decrease with trastuzumab plus chemotherapy, and a 5.52% incidence of LVEF decrease with trastuzumab, pertuzumab, and chemotherapy.

**Meaning:**

Trastuzumab emtansine had the lowest incidence of LVEF decrease, and trastuzumab deruxtecan, trastuzumab plus chemotherapy, and trastuzumab plus pertuzumab plus chemotherapy had similar incidences of LVEF decrease; however, more research regarding their cardiotoxic effects is needed.

## Teaser

This systematic review and meta-analysis compares the incidence of cardiotoxic effects of antibody-drug conjugates vs standard chemotherapy regimens for erb-b2 receptor tyrosine kinase 2 (*ERBB2*)–positive locally advanced or metastatic breast cancer (BC).

## Introduction

Breast cancer (BC) is the most common cancer diagnosis and is the second leading cause of cancer death in women.^1^ Overexpression of erb-b2 receptor tyrosine kinase 2 (*ERBB2*, formerly HER2) denotes an aggressive subtype that comprises 15% to 20% of BCs; it was associated with an increased risk for metastasis and a poor prognosis^2^ before the advent of *ERBB2*-targeted therapies in the contemporary era.^3,4^ Trastuzumab and pertuzumab are anti-*ERBB2* monoclonal antibodies that act synergistically with cytotoxic chemotherapy and have shown improved clinical outcomes in both early stage and metastatic BC^5,6^; however, notable adverse effects of *ERBB2*-targeted therapy are cardiotoxic effects, which are primarily dose independent and largely reversible.^6,7^ They usually manifest as asymptomatic decreases in left ventricular ejection fraction (LVEF), with an incidence ranging from 5% to 19%, but they can manifest as symptomatic heart failure, although the risk is highest in patients receiving concomitant or sequential anthracycline chemotherapy.^8,9^

To mitigate this cardiotoxic effect, novel antibody-drug conjugates (ADCs), such as trastuzumab emtansine and trastuzumab deruxtecan, can be used to treat BC. ADCs contain an *ERBB2*-targeted antibody, a cytotoxic payload, and a chemical linker, with the goal of combining the precision of the antibody with the efficacy of the chemotherapy agent.^10,11^ This precision medicine–based approach aims to more effectively deliver the cytotoxic payload directly to the target antigens on the cancer cells, all while minimizing the systemic toxic effects.^10,11^ Recent phase 3 clinical trials have demonstrated superior progression-free survival, overall survival, and objective response rate with the use of both ADCs compared with conventional regimens in patients who had progression of previously treated locally advanced or metastatic *ERBB2*-positive BC,^12,13,14^ with rates of LVEF decrease between 1% and 12%.^15^

There is a critical need to evaluate the cardiotoxic profiles of these novel ADC agents and compare them with the current standard-of-care regimens for *ERBB2*-positive locally advanced or metastatic BC. Through a single-group meta-analysis of phase 3 clinical trials, we aimed to investigate whether treatment with trastuzumab emtansine or trastuzumab deruxtecan resulted in a lower incidence of LVEF decrease when compared with regimens containing trastuzumab plus chemotherapy or trastuzumab plus pertuzumab plus chemotherapy.

## Methods

### Literature Search Strategy

This meta-analysis and systematic review was conducted in accordance with the Preferred Reporting Items for Systematic Reviews and Meta-Analyses (PRISMA) reporting guidelines.^16^ We performed the systematic literary search in December of 2024 from the time period of 2000 to 2024 and encompassed 4 databases: PubMed, ScienceDirect, Cochrane Library, and ClinicalTrials.gov. The search strategy consisted of keywords and standardized MeSH terms, as well as Boolean operators (“OR” or “AND”). The keywords used to source studies for both groups of the study are summarized in eTable 1 in Supplement 1. This review was not registered, and a protocol was not prepared.

### Inclusion and Exclusion Criteria

Studies were included if they met the following criteria: (1) phase 3 clinical trials that investigated locally advanced or metastatic *ERBB2*-positive BC; (2) clearly defined LVEF decrease or heart failure definitions; (3) clearly defined LVEF monitoring frequency by echocardiography or multigated acquisition scan; (4) included studies consisted solely of either trastuzumab emtansine, trastuzumab deruxtecan, or one of the first-line to fourth-line chemotherapy regimens for unresectable stage IV *ERBB2*-positive BC as per the 2025 National Comprehensive Cancer Network guidelines^17^; and (5) clearly defined cardiovascular eligibility criteria. Only treatment groups including 1 of these regimens were included in the meta-analysis (eTable 2 in Supplement 1).

### Data Extraction and Quality Assessment

Three blinded reviewers (L.S., A.B., and SK) participated in the abstract and full-text screenings. Conflicting decisions were resolved by collaboration among those same 3 reviewers. S.A.H. also assisted to make the final set of included studies robust. For each selected study, the following data were extracted: chemotherapy regimen, study name, trial number, author, year of publication, number of patients, median follow-up, median age, *ERBB2* status of the cohort, hormone receptor status, dose and regimen of chemotherapy, LVEF and/or heart failure assessment, LVEF decrease and/or heart failure definition, and LVEF decrease and/or heart failure incidence. The quality of the included studies was determined by 2 reviewers (L.S. and A.B.) using the Jadad scale (eTable 3 in Supplement 1).^18^ None of the included ADC studies reported heart failure as an outcome; therefore, it is not presented in the results.

### Statistical Analysis

This was a single-group meta-analysis investigating the cardiotoxic effects of chemotherapy regimens containing trastuzumab, in which outcomes from the experimental group of each included study were pooled. We performed the pooled analysis using logit-transformed proportions with the inverse variance method and a DerSimonian-Laird random-effects model^19^ for between-study variance, with Wilson score 95% CIs. We assessed heterogeneity using Cochran *Q* statistic and quantified it using Higgins *I*^2^ statistic.^20^ We assessed for publication bias and small-study effects through visual inspection of funnel plots and statistically through the regression-based Egger test. A trim-and-fill analysis was used if evidence of publication bias was found. A *P* < .05 was considered statistically significant. The results were presented as a pooled analysis in forest plots. R statistical software version 4.4.2 (R Project for Statistical Computing) was used for statistical analysis.^21^

## Results

### Study Characteristics

**Figure 1.**
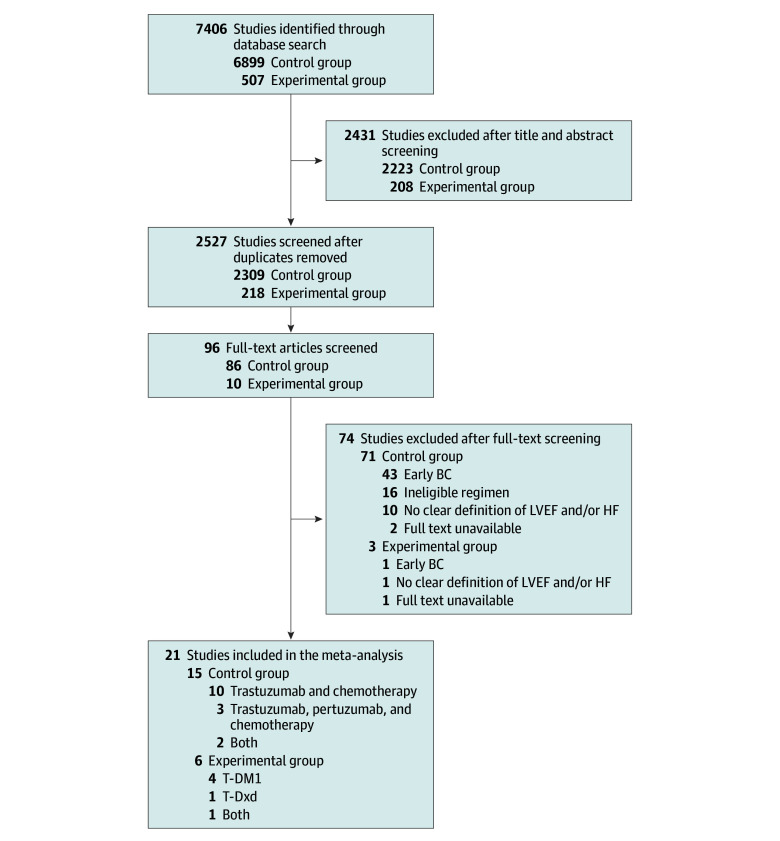
Search Strategy and Study Selection for Both Study Groups BC indicates breast cancer; HF, heart failure; LVEF, left ventricular ejection fraction; T-DM1, trastuzumab emtansine; and T-DXd, trastuzumab deruxtecan.

For the control group, 6899 studies were retrieved from the initial literature search, of which 4590 were duplicates and removed. Of the 2309 remaining studies, 2223 were excluded after abstract screening and 86 were included for full-text screening according to the inclusion criteria. A total of 15 studies were included in the control group of the final meta-analysis.^22,23,24,25,26,27,28,29,30,31,32,33,34,35,36^ Ten studies investigated trastuzumab plus chemotherapy, 3 studies investigated trastuzumab plus pertuzumab plus chemotherapy, and 2 studies investigated both regimens (Figure 1). The trastuzumab plus chemotherapy studies cumulatively involved 2929 patients. The trastuzumab plus pertuzumab plus chemotherapy studies cumulatively involved 2411 patients (Table 1).

**Table 1.** Studies Included in the Meta-Analysis

Chemotherapy regimen and study	No. of patients	Median follow-up, mo	Median age, y	HER2 (*ERBB2*) status of the cohort	Dose and regimen of chemotherapy
T-DM1					
Krop et al,^12^ 2017	404	30.5	53	HER2-positive, unresectable, locally advanced or recurrent BC or MBC who had been previously treated with both trastuzumab and lapatinib in the advanced setting and a taxane in any setting	IV T-DM1 3.6 mg/kg intravenously every 21 d
Montemurro et al,^38^ 2018	2002	20.6	55	HER2-positive recurrent, MBC or unresectable locally advanced BC; patients had received a prior anti-HER2 agent and chemotherapy (prior taxane was not required) and had progressed on or after the most recent treatment or within 6 mo of completing adjuvant therapy	IV T-DM1 3.6 mg/kg intravenously every 3 wk
Perez et al,^24^ 2019	367	54	52	HER2-positive locally advanced BC or MBC	IV T-DM1 3.6 mg/kg body weight every 3 wk
Verma et al,^13^ 2012	495	13	53	Documented progression of unresectable, locally advanced or metastatic HER2-positive BC previously treated with a taxane and trastuzumab	IV T-DM1 3.6 mg/kg body weight every 3 wk
T-DXd					
André et al,^14^ 2023	406	21.5	54.2	Unresectable or MBC that was centrally confirmed as HER2-positive; patient had previously received trastuzumab emtansine and had documented radiological disease progression	IV T-DXd 5.4 mg/kg body weight every 3 wk
Both T-DM1 and T-DXd					
Cortés et al,^37^ 2024	T-DM1, 263; T-DXd, 261	41	T-DM1, 54.3; T-DXd, 54.2	HER2-positive, unresectable or MBC	IV T-DM1 3.6 mg/kg body weight every 3 wk; IV T-DXd 5.4 mg/kg body weight every 3 wk
TC					
Mezzanotte-Sharpe et al,^22^ 2024	48	71.4	55	Histologically confirmed HER2-positive MBC, and no prior systemic therapies were considered for enrollment; prior taxane and trastuzumab were allowed if given >12 mo before recurrence	Paclitaxel 90 mg/m^2^ IV weekly ×3 every 4 wk (6 cycles) or paclitaxel 80 mg/m^2^ IV weekly ×3 every 4 wk plus carboplatin area under the concentration-time curve 2 IV weekly ×3 every 4 wk (6 cycles) with trastuzumab (2 mg/kg IV weekly [after initial loading dose of 4 mg/kg] for 6 cycles)
Rugo et al,^23^ 2021	228	31.1	54	HER2-positive MBC	IV trastuzumab every 3 wk; an initial 8-mg/kg loading dose was administered over 90 min, followed by dosing every 3 wk of 6 mg/kg over 30 min; the choice of taxane (docetaxel or paclitaxel) was by investigator decision at each study site and was applied to all patients enrolled at that site; docetaxel was administered at 75 mg/m^2^ every 3 wk and paclitaxel at 80 mg/m^2^ weekly; paclitaxel could be omitted by investigator choice for 1 wk every 4 wk
Perez et al,^24^ 2019	365	54	55	HER2-positive locally advanced BC or MBC	IV paclitaxel 80 mg/m^2^ weekly or IV docetaxel 75 or 100 mg/m^2^ every 3 wk for 6 cycles; IV trastuzumab was administered at standard doses (with docetaxel: 8 mg/kg loading dose and 6 mg/kg for subsequent cycles; with paclitaxel: 4 mg/kg loading dose and 2 mg/kg in subsequent wk)
Hurvitz et al,^25^ 2015	239	41.3	52	HER2-positive, locally recurrent invasive BC unamenable to resection with curative intent or metastatic disease	IV trastuzumab 4 mg/kg loading dose on d 1 with subsequent weekly doses of 2 mg/kg of each 4-wk cycle; IV paclitaxel at a dose of 80 mg/m^2^ on d 1, 8, and 15 of each 4-wk cycle
Gelmon et al,^26^ 2015	326	21.5	54.4	HER2-positive MBC (stage IV) at primary diagnosis or at relapse after curative intent therapy	IV trastuzumab weekly (loading dose 4 mg/kg, subsequent doses 2 mg/kg) and paclitaxel, 80 mg/m^2^ IV weekly (d 1, 8 and 15 of a 4-wk cycle) or IV trastuzumab weekly (loading dose 8 mg/kg, subsequent doses 6 mg/kg) and docetaxel, 75 mg/m^2^ IV q 3 wk (d 1 of a 3 wk cycle); both followed by trastuzumab, 6 mg/kg IV q 3 wk until disease progression
Li et al,^27^ 2022	355	NR	54	HER2-positive MBC	Weekly trastuzumab (4 mg/kg loading dose on cycle 1 d 1; subsequent doses 2 mg/kg) on d 1, 8, 15, and 22 of each 28-d cycle until at least wk 33 and when given together with paclitaxel; paclitaxel was administered on d 1, 8, and 15 of each 28-d cycle (starting dose 80 mg/m^2^)
Valero et al,^28^ 2010	TH, 131; TCH, 132	NR	TH, 52; TCH, 51	Eligible patients were women who had MBC with amplification of the *HER2* gene by fluorescent in situ hybridization	Patients were randomly assigned to 8 3-wk cycles of treatment with TCH, or with TH; TH consisted of trastuzumab 2 mg/kg over 30 min on d 1, 8, and 15 and docetaxel 100 mg/m^2^ on d 1; TCH consisted of trastuzumab 2 mg/kg over 30 min on d 1, 8, and 15, docetaxel 75 mg/m^2^ on d 1, and carboplatin at area under the serum concentration-time curve 6 mg/mL/min over 30-60 min on d 1; the first dose of trastuzumab consisted of 4 mg/kg over 90 min on d 1 for both the TH and TCH groups; on completion of the 8 cycles, patients received trastuzumab 6 mg/kg alone once every 3 wk until disease progression or cumulative toxic effects
Rugo et al,^29^ 2022	270	20.2	56	Eligible patients were aged ≥18 y with confirmed *ERBB2*-positive BC by local or optional central testing of the most recent biopsy, following 2013 ASCO testing recommendations; patients must have had progressive disease after 2 or more lines of prior *ERBB2*-targeted therapy, including pertuzumab, and 1 to 3 lines of nonhormonal MBC therapy	Investigators chose 1 of 4 chemotherapies (capecitabine, eribulin, gemcitabine, or vinorelbine) for each eligible patient; trastuzumab was given IV at 6 mg/kg (over 30-90 min) on d 1 of each 21-d cycle after a loading dose of 8 mg/kg (over 90 min); capecitabine was given orally at 1000 mg/m^2^ twice daily for 14 d followed by 7 d off; eribulin, gemcitabine, and vinorelbine were given IV before antibody infusion at 1.4 mg/m^2^, 1000 mg/m^2^, and 25 to 30 mg/m^2^, respectively, on d 1 and 8 of each cycle.
Urruticoechea et al,^30^ 2017	218	28.6	55	Pathologically confirmed BC; documented MBC; centrally confirmed HER2-positive disease (immunohistochemistry triple positive and/or fluorescence or chromogenic in situ hybridization positive)	IV trastuzumab was administered as an 8-mg/kg loading dose in cycle 1 followed by 6-mg/kg maintenance doses once every 3 wk; capecitabine was administered as an oral 1250 mg/m^2^ dose twice daily (2 wk on, 1 wk off every 3 wk)
Inoue et al,^31^ 2009	Sequential, 49; combination, 50	NR	Sequential, 57.5; combination, 54.3	HER2-positive BC (triple positive on immunohistochemical analysis or gene amplification by fluorescence in situ hybridization–positive as determined by the local institution) confirmed in the primary lesion(s) (in the case of bilateral BC, both right and left lesions) or in the target metastatic lesion(s)	Patients were randomly assigned to 1 of 2 groups: initial treatment with trastuzumab alone, followed by combination therapy with trastuzumab and docetaxel after disease progression, or initial combination therapy with trastuzumab and docetaxel; in the first group, trastuzumab was administered weekly with a starting dose of 4 mg/kg followed by 2 mg/kg as the second and subsequent doses, and docetaxel 60 mg/m^2^ was administered every 3 wk following disease progression
TPC					
Woodward et al,^32^ 2019	50	50	52.9	HER2-positive MBC	3-wk Cycles of IV pertuzumab (840 mg first dose; subsequent doses of 420 mg) and subcutaneous trastuzumab 600 mg; taxane (docetaxel, paclitaxel or nab-paclitaxel) treatment regimen will be determined by the investigator
Miles et al,^33^ 2021	1436	68.7	54	HER2-positive locally recurrent or MBC not amenable to curative resection	Choice of taxane (docetaxel, paclitaxel or nab-paclitaxel) was at the investigators’ discretion; administered weekly or every 3 wk in accordance with recognized guidelines and/or local prescribing information; IV pertuzumab every 3 wk at a dose of 840 mg in cycle 1 followed by 420 mg in subsequent cycles plus IV trastuzumab every 3 wk at a dose of 8 mg/kg in cycle 1 followed by 6 mg/kg for subsequent cycles
Kuemmel et al,^34^ 2021	396	27	NR	Eligible patients were aged ≥18 y with histologically or cytologically confirmed HER2-positive MBC previously untreated with systemic nonhormonal anticancer therapy; prior treatment with ≤2 lines of hormonal therapy, one of which could be in combination with everolimus, was permitted; hormonal therapy concomitant with the use of pertuzumab IV and trastuzumab IV was permitted after chemotherapy discontinuation	All received ≥1 subcutaneous trastuzumab 600 mg fixed dose plus intravenous pertuzumab (loading dose: 840 mg/kg; maintenance: 420 mg/kg) and docetaxel (≥6 cycles; initial dose 75 mg/m^2^) every 3 wk
Both TC and TPC					
Swain et al,^35^2013	TC, 397; TPC, 407	TC, 50.6; TPC, 49.5	NR	HER2-positive MBC who had received no more than one hormonal treatment for metastatic disease; adjuvant or neoadjuvant chemotherapy with or without trastuzumab was allowed	Study drugs were administered intravenously on a 3-wk schedule; patients received pertuzumab or placebo at an initial dose of 840 mg, followed by 420 mg; trastuzumab was given at an initial dose of 8 mg/kg, followed by 6 mg/kg; docetaxel (Taxotere, Sanofi-Aventis) was administered at 75 mg/m^2^
Xu et al,^36^ 2020	TC, 121; TPC, 122	TC, 13.1; TPC, 13.7	TC, 53; TPC, 51	HER2-positive locally recurrent or MBC	Trastuzumab group (8 mg/kg loading dose, then 6 mg/kg every 3 wk) plus docetaxel (75 mg/m^2^ every 3 wk); pertuzumab plus trastuzumab group: pertuzumab 840 mg loading dose, followed by 420 mg every 3 wk) plus trastuzumab (8 mg/kg loading dose, then 6 mg/kg every 3 wk) plus docetaxel (75 mg/m^2^ every 3 wk)

Abbreviations; ASCO, American Society for Clinical Oncology; BC, breast cancer; *ERBB2*, erb-b2 receptor tyrosine kinase 2; HER2, human epidermal growth receptor factor 2; IV, intravenous; MBC, metastatic breast cancer; NR, not reported; TC, trastuzumab plus chemotherapy; TCH, docetaxel, carboplatin, and trastuzumab; T-DM1, trastuzumab emtansine; T-DXd, trastuzumab deruxtecan; TH, docetaxel and trastuzumab; TPC, trastuzumab plus pertuzumab plus chemotherapy.

For the experimental group, a total of 507 studies were retrieved from the initial literature search, of which 289 were duplicates and removed. Of the 218 remaining studies, 208 were excluded after abstract screening and 10 were included for full-text screening on the basis of the inclusion criteria. A total of 6 studies were included in the experimental group of the final meta-analysis.^12,13,14,24,37,38^ Four studies investigated trastuzumab emtansine, 1 study investigated trastuzumab deruxtecan, and 1 study investigated both drugs (Figure 1). The trastuzumab emtansine studies cumulatively involved 3531 patients (Table 1). The trastuzumab deruxtecan studies cumulatively involved 667 patients. For both groups of the study, the *ERBB2* status of the cohort, chemotherapy regimen (Table 1), and LVEF and/or heart failure assessment and incidence are summarized (Table 2).

**Table 2.** LVEF and HF Assessment and Incidence and Inclusion and Exclusion

Chemotherapy regimen and study	Relevant cardiovascular inclusion criteria	LVEF and HF assessment	LVEF decrease and HF definition
T-DM1			
Krop et al,^12^ 2017	LVEF ≥50% by echocardiogram or MUGA scan	LVEF was measured by echocardiogram (preferred method) or MUGA at screening, 6 wk (ie, end of cycle 2), every 12 wk thereafter until study discontinuation, and 30 d after the last treatment dose	LVEF decrease, EF <50% with a ≥15–percentage point decrease from the baseline; HF, NR
Montemurro et al,^38^ 2018	LVEF ≥50% by either echocardiogram or MUGA	LVEF assessments will be performed within 28 d of enrollment, on d 21 (or −7 d) of the cycle for cycle 1, on d 21 (or −7 d) of cycle 3 and every third cycle thereafter, by either echocardiogram or MUGA scan (with echocardiogram as the preferred method)	LVEF decrease, LVEF decrease from the baseline to <40%; HF, NR
Perez et al,^24^ 2019	Excluded: inadequate LVEF at baseline, as defined as LVEF <50% by either echocardiogram or MUGA; history of symptomatic CHF grade ≥3 per NCI CTCAE (version 4.0, appendix 13) or Class ≥II NYHA criteria (appendix 14); history of a decrease in LVEF to <40% or symptomatic CHF with prior trastuzumab treatment	Echocardiogram or MUGA performed at baseline, once on d 15 to 21 of cycle 1 cycle 3, and every third cycle thereafter; an additional assessment was performed at least 28 d after the last dose of study drug	LVEF decrease, EF <50% with a ≥15–percentage point decrease from the baseline; HF, NR
Verma et al,^13^ 2012	Cardiac EF ≥50% by either echocardiogram or MUGA scan	Echocardiogram or MUGA scanning at baseline, 6 wk, 12 wk, and every 12 wk thereafter until discontinuation of the study treatment; an additional assessment was performed 30 d after the last dose of the study drug	LVEF decrease, EF <50% with a ≥15–percentage point decrease from the baseline; HF, NR
T-DXd			
André et al,^14^ 2023	Patients with history of symptomatic CHF (NYHA Class II to IV) and LVEF <50% within 28 d prior to randomization were excluded	Every 4 cycles (±7 d) after cycle 1, perform an echocardiogram or MUGA (note: the same test must be used for the patient throughout the study)	LVEF decrease, grade 2: resting EF 50%-40%; 10%-19% drop from baseline; grade 3: resting EF 39%-20%; ≥20% drop from baseline; grade 4: resting EF <20%; HF, NR
Both T-DM1 and T-DXd			
Cortés et al,^37^ 2024	Patients with history of symptomatic CHF (NYHA class II-IV) and LVEF <50% within 28 d prior to randomization were excluded	Echocardiogram or MUGA every 4 cycles (±7 d) after cycle 1	LVEF decrease, grade 2: resting EF: 40%-50%, 10%-19% decrease from baseline; grade 3: resting EF: 20%-39%, >20% decrease from baseline; HF, NR
TC			
Mezzanotte-Sharpe et al,^22^ 2024	MUGA scan or echocardiogram within 6 wk prior to randomization with an LVEF above the institutional LLN	Echocardiogram or MUGA at baseline, every 3 mo, and 3 mo after treatment	LVEF decrease, symptomatic decline in LVEF to below the LLN or symptomatic diastolic dysfunction; HF, NR
Rugo et al,^23^ 2021	LVEF within institutional range of normal as measured by MUGA scan or echocardiogram	LVEF assessment every 12 wk	LVEF decrease, LVEF decrease below 50%; HF, NR
Perez et al,^24^ 2019	Patients with inadequate LVEF at baseline, as defined as LVEF <50% by either echocardiogram or MUGA, those with history of symptomatic CHF (grade ≥3 per NCI CTCAE version 4.0, appendix 13) or class ≥II NYHA criteria (appendix 14), and those with history of a decrease in LVEF to <40% or symptomatic CHF with prior trastuzumab treatment were excluded	Echocardiogram or MUGA performed at baseline, once on days 15 to 21 of cycle 1 cycle 3, and every third cycle thereafter; an additional assessment was performed at least 28 d after the last dose of study drug	LVEF decrease, EF <50% with a ≥15–percentage point decrease from the baseline; HF, NR
Hurvitz et al,^25^ 2015	LVEF value at LLN or higher within 4 wk of randomization	Echocardiogram every 12 wk	LVEF decrease, grade 1: asymptomatic, resting EF; <60% to 50%; shortening fraction; <30% to 24%; grade 2: asymptomatic, resting; EF <50% to 40%; shortening fraction <24% to 15%; grade 3: symptomatic CHF responsive; to intervention; EF <40% to 20% shortening fraction <15%; HF, NR
Gelmon et al,^26^ 2015	Baseline LVEF ≥50% (determined by echocardiography or MUGA scanning)	Echocardiogram or MUGA every 12 wk during treatment, 4 wk after treatment	LVEF decrease, absolute decrease in LVEF of ≥20%; measured by echocardiogram or MUGA; HF, NR
Li et al,^27^ 2022	LVEF within institutional range of normal, measured by either 2-dimensional echocardiogram or MUGA scan	Echocardiogram or MUGA as per local standard of care	LVEF decrease, absolute decline in LVEF ≥10% and below the LLN; HF, NR
Valero et al,^28^ 2010	LVEF normal by MUGA or echocardiogram	Echocardiogram or MUGA within 1 mo of study entry, then every 4 mo	LVEF decrease, absolute LVEF decline >15%; HF, NR
Rugo et al,^29^ 2022	Patients with LVEF <50% by echocardiogram or MUGA scan were excluded	LVEF was monitored every 6 wk for 24 wk and then every 12 wk thereafter by MUGA or echocardiogram	LVEF decrease, ≥16% absolute decrease in LVEF from pretreatment values, LVEF below institutional normal limits and ≥10% absolute decrease in LVEF from pretreatment values; HF, NR
Urruticoechea et al,^30^ 2017	LVEF ≥50% at screening period to be determined by either echocardiogram or MUGA scan (with echocardiogram as the preferred method)	Every 3 cycles (9 wk)	LVEF decrease, asymptomatic left ventricular systolic dysfunction; includes asymptomatic LVEF drop of ≥10 percentage points below baseline and value, 50%; asymptomatic LVEF drop that required treatment or that led to treatment discontinuation; HF, NR
Inoue et al,^31^ 2009	LVEF >50% on echocardiography	NR	LVEF decrease, LVEF <50%; HF, NR
TPC			
Woodward et al,^32^ 2019	LVEF of ≥50% measured by echocardiogram or MUGA scan before the first doses of pertuzumab and trastuzumab	LVEF assessment every 12 wk	LVEF decrease, LVEF decrease below 50%; HF, NYHA II-III
Miles et al,^33^ 2021	LVEF of at least 50%	Echocardiogram or MUGA every 3 treatment cycles	LVEF decrease, EF <50%, >10% drop from baseline; HF, NYHA I-IV
Kuemmel et al,^34^ 2021	Baseline LVEF ≥50%	LVEF will be assessed during the screening period within 6 wk prior to first dose of study drug and every 3 treatment cycles by either echocardiogram or MUGA scan (with echocardiogram as the preferred method)	LVEF decrease, LVEF <50% and decrease ≥10% points from baseline; HF, NR
Both TC and TPC			
Swain et al,^35^ 2013	LVEF ≥50% at baseline (within 42 d of randomization)	Echocardiogram or MUGA at baseline, every 9 wk during study treatment, at treatment discontinuation, every 6 mo in the first year after discontinuation, and annually thereafter for up to 3 y	LVEF decrease, LVEF decrease below 50% and decrease from baseline ≥10%; HF, grade 2: asymptomatic, resting EF <50% to 40%; SF <24% to 15%; grade 3: LVSD was defined as either symptomatic CHF responsive to intervention, or LVEF 20%-39%; grade 4: LVSD was defined as refractory CHF or poorly controlled; or LVEF 20%; or intervention such as ventricular assist device, ventricular reduction surgery, or heart transplant indicated
Xu et al,^36^ 2020	LVEF ≥55% at baseline (within 42 d of randomization)	Echocardiogram or MUGA at screening, baseline, every 9 wk from randomization until the treatment discontinuation visit (or more frequently as needed), every 6 mo thereafter for the first year, and annually for up to 3 y until the end of the study.	LVEF decrease, LVEF decrease below 50% and decrease from baseline ≥10%; HF, grade 1: asymptomatic with laboratory (eg, B-natriuretic peptide) or cardiac imaging abnormalities; grade 2: symptoms with moderate activity or exertion; grade 3: symptoms at rest or with minimal activity or exertion; hospitalization; new onset of symptoms; grade 4: life-threatening consequences; urgent intervention indicated (eg, continuous IV therapy or mechanical hemodynamic support)

Abbreviations: CHF, congestive heart failure; CTCAE, Common Terminology for Adverse Events; EF, ejection fraction; HER2, human epidermal growth receptor factor 2; HF, heart failure; IV, intravenous; LLN, lower limit of normal; LVEF, left ventricular ejection fraction; LVSD, left ventricular systolic dysfunction; MUGA, multigated acquisition; NCI, National Cancer Institute; NR, not reported; NYHA, New York Health Association; TC, trastuzumab plus chemotherapy; T-DM1, trastuzumab emtansine; T-DXd, trastuzumab deruxtecan; TPC, trastuzumab plus pertuzumab plus chemotherapy.

### Outcomes

**Figure 2.**
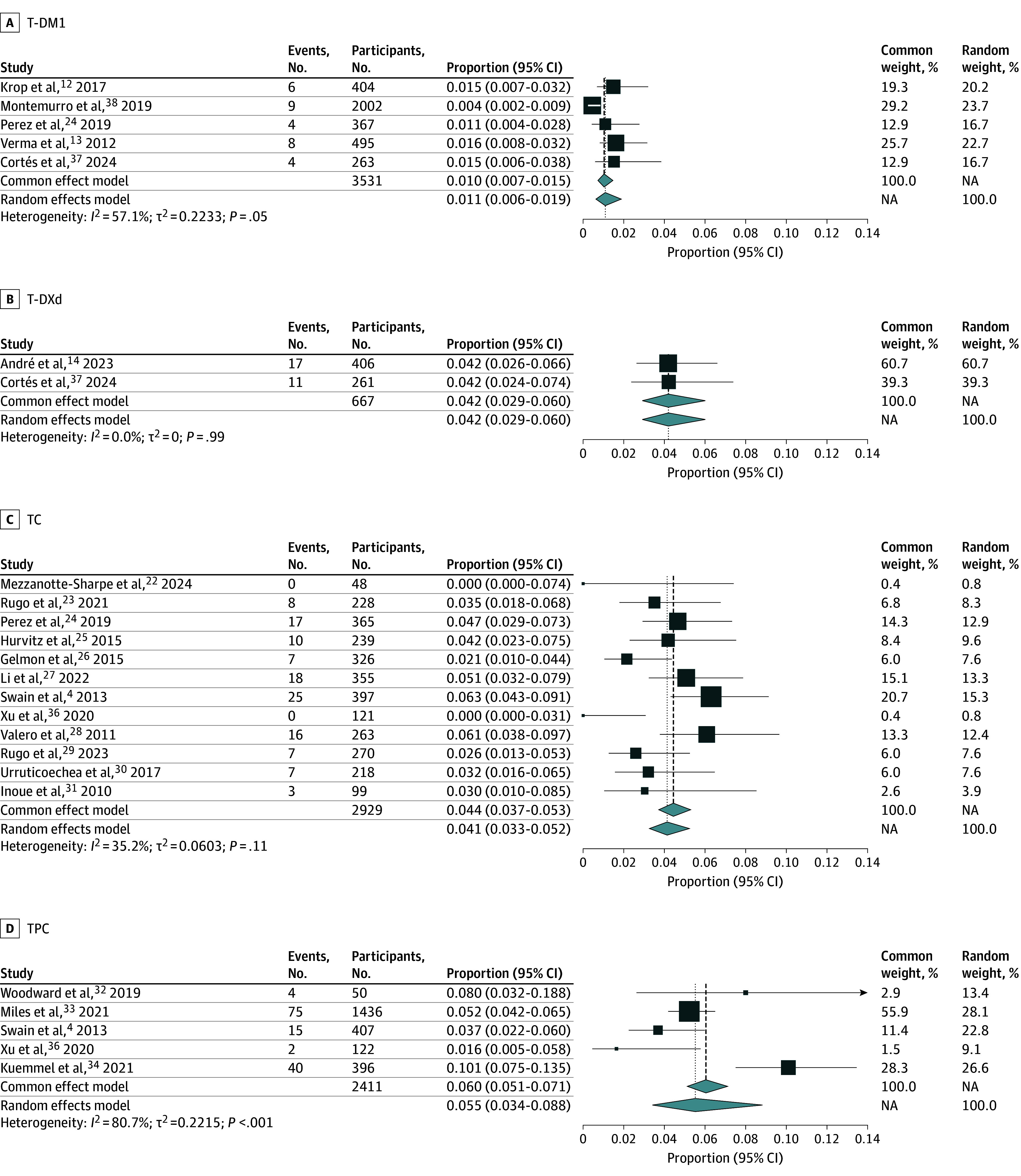
Forest Plots Summarizing the Incidence of Left Ventricular Ejection Fraction Decrease Plots show data for trastuzumab emtansine (T-DM1; A), trastuzumab deruxtecan (T-DXd; B), trastuzumab plus chemotherapy (TC; C), and trastuzumab plus pertuzumab plus chemotherapy (TPC; D). NA indicates not applicable. The size of each square is proportional to the weight of the study in the meta-analysis, and the width of the diamond reflects the 95% CI of the pooled effect estimate.

A pooled random effects model investigating the incidence of LVEF decrease across 4 different chemotherapy regimens is summarized (Table 3). Most studies (3 of 6 in the experimental group and 9 of 15 in the control group) reported a common definition of LVEF decrease defined as a decrease in ejection fraction below 50% and/or a 10% to 15% or higher decrease from baseline. The rest of the studies had similar definitions for LVEF decrease and are summarized in Table 2. Pooled results from the 5 studies evaluating trastuzumab emtansine demonstrated a 1.09% (95% CI, 0.63%-1.88%) incidence of LVEF decrease (Figure 2A). Visual inspection of the funnel plot showed a risk of publication bias, so a trim-and-fill approach was used to adjust for publication bias (eFigure 1A in Supplement 1). A trim-and-fill random effects model (eFigure 2A in Supplement 1) added 1 study and demonstrated a 0.94% (95% CI, 0.56%-1.57%) incidence of LVEF decrease. Pooled results from the 2 studies evaluating trastuzumab deruxtecan demonstrated a 4.20% (95% CI, 2.91%-6.01%) incidence of LVEF decrease (Figure 2B). Visual inspection of the funnel plot for trastuzumab deruxtecan did not demonstrate an obvious risk for publication bias (eFigure 1B in Supplement 1). Pooled results from the 12 studies evaluating trastuzumab plus chemotherapy demonstrated a 4.14% (95% CI, 2.26%-5.23%) incidence of LVEF decrease (Figure 2C). Visual inspection of the funnel plot showed a risk of publication bias, so a trim-and-fill approach was used to adjust for publication bias (eFigure 1C in Supplement 1). A trim-and-fill random effects model (eFigure 2B in Supplement 1) added 5 studies and demonstrated a 4.85% (95% CI, 3.73%-6.28%) incidence of LVEF decrease. Pooled results from the 5 studies evaluating trastuzumab plus pertuzumab plus chemotherapy demonstrated a 5.52% (95% CI, 3.41%-8.83%) incidence of LVEF decrease (Figure 2D). Visual inspection of the funnel plot for trastuzumab plus pertuzumab plus chemotherapy did not demonstrate an obvious risk for publication bias (eFigure 1D in Supplement 1).

**Table 3.** Incidence of Left Ventricular Ejection Fraction Decrease Summarized for the Various Chemotherapy Regimens

Regimen	No. of cases	Total No. of patients	No. of studies	Incidence, % (95% CI)	*I*^2^, %	*P* value
Trastuzumab emtansine	31	3531	5	1.09 (0.63-1.88)	57.1	.05
Trastuzumab deruxtecan	28	667	2	4.20 (2.91-6.01)	0.0	.99
Trastuzumab plus chemotherapy	118	2929	12	4.14 (2.26-5.23)	35.2	.11
Trastuzumab plus pertuzumab plus chemotherapy	136	2411	5	5.52 (3.41-8.83)	80.7	<.001

## Discussion

This single-group, systematic review and meta-analysis reported the pooled incidence of LVEF decrease across 4 different chemotherapy regimens that contained trastuzumab. When accounting for publication bias, we found that trastuzumab emtansine had the lowest incidence of LVEF decrease of 0.94%, whereas trastuzumab deruxtecan, trastuzumab plus chemotherapy, and trastuzumab plus pertuzumab plus chemotherapy all had similar incidences of LVEF decrease ranging between 4.20% and 5.52%. To our knowledge, this is the first study that compares the incidence of LVEF decrease of the novel ADCs with standard-of-care trastuzumab and pertuzumab regimens in phase 3 clinical trials, albeit indirectly. This is an important comparison because ADCs like trastuzumab emtansine and trastuzumab deruxtecan have a favorable safety profile and have shown promise in efficacy as a therapy for previously treated *ERBB2*-positive BC that has progressed on standard-of-care regimens, but there is a paucity of data comparing the safety profile and clinical efficacy among ADCs, the safety profile and clinical efficacy of ADCs vs the standard-of-care trastuzumab-containing chemotherapy regimens, and the potential of these agents to be used as a first-line therapy for both early-stage and metastatic *ERBB2*-positive BC.

The phase 3 clinical trial, MARIANNE, demonstrated that trastuzumab emtansine had noninferior progression-free survival, similar overall survival, and a lower incidence of grade 3 or higher adverse events compared with a taxane and trastuzumab regimen. However, the study did not compare trastuzumab emtansine with the current standard of care trastuzumab, pertuzumab, and taxane regimen.^24^ To our knowledge, MARIANNE is the only phase 3 clinical trial that investigated trastuzumab emtansine as a first-line agent for *ERBB2*-positive metastatic BC.^24^ DESTINY-Breast09 is an ongoing phase 3 clinical trial investigating the therapeutic potential of trastuzumab deruxtecan as a first-line agent in *ERBB2*-positive metastatic BC.^39^ Interim results from the trial demonstrated that trastuzumab deruxtecan plus pertuzumab had statistically significant and clinically meaningful improvement in progression-free survival when compared with trastuzumab plus pertuzumab plus taxane (40.7 months vs 26.9 months; hazard ratio, 0.56; 95% CI, 0.44-0.71; *P* < .00001), although the trastuzumab deruxtecan plus placebo group remains blinded until final progression-free survival analysis.^40^ DESTINY-BREAST03 is the only phase 3 clinical trial to directly compare trastuzumab emtansine vs trastuzumab deruxtecan,^37^ highlighting the need for additional clinical trials comparing the safety profile and clinical efficacy between ADCs. Trastuzumab deruxtecan has also shown promise in previously treated *ERBB2*-low metastatic BC, as both the DESTINY-BREAST04 and DESTINY-BREAST06 phase 3 clinical trials demonstrated longer progression-free survival after treatment with trastuzumab deruxtecan vs the physician’s choice of chemotherapy.^41,42^

The lower incidence of cardiotoxic effects observed in our meta-analysis with trastuzumab emtansine could be explained by the lack of the bystander effect with this ADC in contrast to trastuzumab deruxtecan, which does exhibit the bystander effect. In contrast to trastuzumab emtansine, the bystander effect, along with the membrane permeability of trastuzumab deruxtecan, allows it to cross cell membranes and exhibit cytotoxic effects on surrounding tumor cells, irrespective of *ERBB2* levels, and has shown promise in treating *ERBB2* low or heterogenous and trastuzumab emtansine–refractory cancers.^43,44^ The bystander effect’s ability to maximize cytotoxic effects at the expense of off-target adverse effects could explain the results of DESTINY-BREAST03, which found superior clinical efficacy in patients treated with trastuzumab deruxtecan compared with trastuzumab emtansine, but also found a higher number of adverse events associated with drug discontinuation, dose reduction, and drug interruption with trastuzumab deruxtecan than with trastuzumab emtansine.^37^ These findings are consistent with the findings of our meta-analysis, which found an LVEF incidence of 0.94% with trastuzumab emtansine use vs 4.20% with trastuzumab deruxtecan use.

The strength of this meta-analysis is the inclusion criteria that investigated studies with a clear definition of LVEF decrease based on a decrease in ejection fraction from baseline. This eliminated the ambiguity in making assumptions on what qualified as LVEF decrease across studies and allowed us to more accurately compare the incidence across different chemotherapy regimens. Another strength is that all the studies included were phase 3 clinical trials that employed ADCs or standard-of-care trastuzumab-containing regimens as per the National Comprehensive Cancer Network guidelines for *ERBB2*-positive BC. This allowed for the inclusion of regimens that had the most relevance to clinical practice.

### Limitations

A major limitation of this meta-analysis is that it did not allow for direct comparison in the incidence of LVEF decrease between ADCs and standard-of-care regimens, as only 1 study we included investigated this.^24^ The results from that study are similar to the findings of our study; MARIANNE also found a lower incidence of LVEF decrease in their trastuzumab emtansine cohort compared with their trastuzumab plus chemotherapy cohort. This limitation has been addressed above as a future direction for additional clinical trials, such as DESTINY-Breast09, to investigate the potential of ADCs as first-line therapy for *ERBB2*-positive metastatic BC with direct comparison to trastuzumab-containing standard regimens with respect to their clinical efficacy and safety profile. This meta-analysis was conducted using study-level data, which limited our ability to account for differences in baseline characteristics across trials, such as age, race, ethnicity, and trial site experience. Although randomization within each included study minimizes confounding, differences across studies may have contributed to the observed results. The median age of participants in the trials is in the mid 50s, which is younger than the community population and may lead to underestimation of the true extent of cardiotoxicity in the community population. Statistical modeling will not fully represent a real-world population and remains an inherent limitation of meta-analyses. Additionally, only 2 studies included investigated the incidence of LVEF decrease in the trastuzumab deruxtecan, which limits statistical power and makes the results more susceptible to the influence of potential heterogeneity. The number of patients in each cohort who received prior anthracyclines was not known; as a result, we were unable to evaluate how anthracycline use impacts the incidence of LVEF dysfunction. In addition, some studies included more patients with prior exposure to *ERBB2*-targeted therapies in the pretreatment phase, which may have increased their susceptibility to subsequent cardiotoxic effects in the future. Although many studies reported a similar definition of LVEF decrease, the lack of a standardized definition of LVEF decrease across studies has the potential to introduce bias to the analysis. Another limitation is that secondary cardiovascular outcomes, such as atrial fibrillation, myocardial infarction, and coronary artery disease, were not reported in the text of the studies included in the meta-analysis. Although we applied a logit transformation to account for rare events, our use of the DerSimonian-Laird estimator has limitations, as it may underestimate variability when event rates are very low owing to the method’s approximation of the within-study variability of the proportion by a normal distribution.^45^

## Conclusions

This single-group systematic review and meta-analysis that indirectly compared the incidence of LVEF decrease across 4 chemotherapy regimens containing trastuzumab found that trastuzumab emtansine had the lowest incidence of LVEF decrease; trastuzumab deruxtecan, trastuzumab plus chemotherapy, and trastuzumab plus pertuzumab plus chemotherapy had similar incidences of LVEF decrease. Trastuzumab emtansine and trastuzumab deruxtecan have shown promise in treating *ERBB2*-positive metastatic BC; however, there is a paucity of data comparing the cardiotoxic effects among ADCs, of ADCs vs the standard-of-care trastuzumab-containing chemotherapy regimens, and the potential of ADCs as first-line therapy for both early-stage and metastatic BC.

## Notes

Supplement 1. eTable 1. Keywords used in the database search to source articles for inclusion in the meta-analysis. eTable 2. Regimens included in the control arm of the meta-analysis eTable 3. Jadad Scale for Reporting Randomized Controlled Trials. eFigure 1. Funnel plots summarizing the risk of publication bias for a) T-DM1, b) T-DXd, c) TC, d) TPC. eFigure 2. Trim and fill approach adjusting for publication bias

Supplement 2. Data Sharing Statement
