# Supplementary material for: Cardiotoxic Effects of Antibody Drug Conjugates vs Standard Chemotherapy in ERBB2-Positive Advanced Breast Cancer: A Systematic Review and Meta-Analysis
Source: JAMA Netw Open. 2025 Nov 9;8(11):e2540336. doi: 10.1001/jamanetworkopen.2025.40336 (PMC12598513; doi:10.1001/jamanetworkopen.2025.40336)
Supplement: Supplement 1. — eTable 1. Keywords used in the database search to source articles for inclusion in the meta-analysis. eTable 2. Regimens included in the control arm of the meta-analysis eTable 3. Jadad Scale for Reporting Randomized Controlled Trials. eFigure 1. Funnel plots summarizing the risk of publication bias for a) T-DM1, b) T-DXd, c) TC, d) TPC. eFigure 2. Trim and fill approach adjusting for publication bias [file jamanetwopen-e2540336-s001.pdf]

Supplemental Online Content

Seth L, Bhave A, Kollapaneni S, et al. Cardiotoxic effects of antibody drug conjugates vs standard chemotherapy in ERBB2-positive advanced breast cancer: a systematic review and meta-analysis. *JAMA Netw Open*. 2025;8(10):e2540336. doi:10.1001/jamanetworkopen.2025.40336

eTable 1. Keywords used in the database search to source articles for inclusion in the meta-analysis.
eTable 2. Regimens included in the control arm of the meta-analysis
eTable 3. Jadad Scale for Reporting Randomized Controlled Trials.
eFigure 1. Funnel plots summarizing the risk of publication bias for a) T-DM1, b) T-DXd, c) TC, d) TPC.
eFigure 2. Trim and fill approach adjusting for publication bias

This supplemental material has been provided by the authors to give readers additional information about their work.

© 2025 Seth L et al. *JAMA Network Open*.

eTable 1. Keywords used in the database search to source articles for inclusion in the meta-analysis.

Arm	Keywords	Search
**Experimental**	#1: "trastuzumab emtansine" OR "trastuzumab-emtansine" OR "T-DM1" OR "TDM-1" OR "trastuzumab-DM1" OR "trastuzumab DM1" OR "Kadcyla" OR "Ado-trastuzumab" OR "trastuzumab deruxtecan" OR "trastuzumab-deruxtecan" OR "DS-8201a" OR "T-DXd" OR "DS-8201" OR "Enhertu" OR "fam-trastuzumab deruxtecan-nxki"<br><br>#2: "heart failure" OR "cardiac failure" OR "atrial fibrillation" OR "myocardial infarction" OR "myocardial ischemia" OR "cardiac ischemia" OR "cardiomyopathy" OR "coronary artery disease" OR "ischemic heart disease" OR "heart disease" OR "ventricular dysfunction" OR "ejection fraction decreased" OR "decreased ejection fraction" OR "systolic dysfunction"	#1 AND #2
**Control**	#1: "trastuzumab" OR "pertuzumab" OR "herceptin" OR "perjeta"<br><br>#2: "heart failure" OR "cardiac failure" OR "atrial fibrillation" OR "myocardial infarction" OR "myocardial ischemia" OR "cardiac ischemia" OR "cardiomyopathy" OR "coronary artery disease" OR "ischemic heart disease" OR "heart disease" OR "ventricular dysfunction" OR "ejection fraction decreased" OR "decreased ejection fraction" OR "systolic dysfunction"	#1 AND #2

© 2025 Seth L et al. *JAMA Network Open*.

eTable 2. Regimens included in the control arm of the meta-analysis

**Chemotherapy Regimen**
• Pertuzumab + trastuzumab + docetaxel<br>• Pertuzumab + trastuzumab + paclitaxel<br>• Trastuzumab + docetaxel<br>• Trastuzumab + paclitaxel +/- carboplatin<br>• Trastuzumab + tucatinib + capecitabine<br>• Trastuzumab + vinorelbine<br>• Trastuzumab + capecitabine<br>• Trastuzumab + gemcitabine<br>• Trastuzumab + eribulin<br>• Trastuzumab + cyclophosphamide<br>• Trastuzumab + Ixabepilone<br>• Trastuzumab + CMF (cyclophosphamide/methotrexate/fluorouracil)<br>• Trastuzumab + docetaxel + capecitabine<br>• Trastuzumab + gemcitabine + paclitaxel<br>• Trastuzumab + gemcitabine + carboplatin

© 2025 Seth L et al. *JAMA Network Open*.

eTable 3. Jadad Scale for Reporting Randomized Controlled Trials.

**Chemotherapy regimen**	**Study name**	**Author and year**	**Jadad Score**
**Trastuzumab emtansine studies**			
	Trastuzumab emtansine versus treatment of physician's choice in patients with previously treated HER2-positive metastatic breast cancer (TH3RESA): final overall survival results from a randomised open-label phase 3 trial	Krop et al 2017	3
	Safety of trastuzumab emtansine (T-DM1) in patients with HER2-positive advanced breast cancer: Primary results from the KAMILLA study cohort 1	Montemurro et al 2018	1
	Trastuzumab Emtansine With or Without Pertuzumab Versus Trastuzumab With Taxane for Human Epidermal Growth Factor Receptor 2–Positive Advanced Breast Cancer: Final Results From MARIANNE	Perez et al 2019	3
	Trastuzumab Emtansine for HER2-Positive Advanced Breast Cancer EMILIA	Verma et al 2012	3
**Trastuzumab deruxtecan studies**			
	Trastuzumab deruxtecan versus treatment of physician's choice in patients with HER2-positive metastatic breast cancer (DESTINY-Breast02): a randomised, open-label, multicentre, phase 3 trial	André et al 2023	3
**Both**			
	Trastuzumab deruxtecan versus trastuzumab emtansine in HER2-positive metastatic breast cancer: long-term survival analysis of the DESTINY-Breast03 trial	Cortés et al 2024	3
**Trastuzumab + chemotherapy studies**			
	A randomized phase III double-blind placebo-controlled trial of first-line chemotherapy and trastuzumab with or without bevacizumab for patients with HER2/neu-positive metastatic breast cancer: a trial of the ECOG-ACRIN Cancer Research Group (E1105)	Mezzanotte-Sharpe et al 2024	5
	Final overall survival analysis of the phase 3 HERITAGE study demonstrates equivalence of trastuzumab-dkst to trastuzumab in HER2-positive metastatic breast cancer	Rugo et al 2021	5
	Trastuzumab Emtansine With or Without Pertuzumab Versus Trastuzumab With Taxane for Human Epidermal Growth Factor Receptor 2–Positive Advanced Breast Cancer: Final Results From MARIANNE	Perez et al 2019	3
	Combination of everolimus with trastuzumab plus paclitaxel as first-line treatment for patients with HER2-positive advanced breast	Hurvitz et al 2015	5

© 2025 Seth L et al. *JAMA Network Open*.

	cancer (BOLERO-1): a phase 3, randomised, double-blind, multicentre trial		
	Lapatinib or Trastuzumab Plus Taxane Therapy for Human Epidermal Growth Factor Receptor 2–Positive Advanced Breast Cancer: Final Results of NCIC CTG MA.31	Gelmon et al 2015	3
	Long-Term Safety and Effectiveness of PF-05280014 (a Trastuzumab Biosimilar) Treatment in Patients with HER2-Positive Metastatic Breast Cancer: Updated Results of a Randomized, Double-Blind Study	Li et al 2022	5
	Multicenter phase III randomized trial comparing docetaxel and trastuzumab with docetaxel, carboplatin, and trastuzumab as first-line chemotherapy for patients with HER2-gene-amplified metastatic breast cancer (BCIRG 007 study): two highly active therapeutic regimens	Valero et al 2011	3
	Margetuximab Versus Trastuzumab in Patients With Previously Treated HER2-Positive Advanced Breast Cancer (SOPHIA): Final Overall Survival Results From a Randomized Phase 3 Trial	Rugo et al 2022	5
	Randomized Phase III Trial of Trastuzumab Plus Capecitabine With or Without Pertuzumab in Patients With Human Epidermal Growth Factor Receptor 2–Positive Metastatic Breast Cancer Who Experienced Disease Progression During or After Trastuzumab-Based Therapy	Urruticoechea et al 2017	3
	Randomized phase III trial of trastuzumab monotherapy followed by trastuzumab plus docetaxel versus trastuzumab plus docetaxel as first-line therapy in patients with HER2-positive metastatic breast cancer: the JO17360 Trial Group	Inoue et al 2009	3
**Trastuzumab + pertuzumab + chemotherapy studies**			
	Results From the First Multicenter, Open-label, Phase IIIb Study Investigating the Combination of Pertuzumab With Subcutaneous Trastuzumab and a Taxane in Patients With HER2-positive Metastatic Breast Cancer (SAPPHIRE)	Woodward et al 2019	1

© 2025 Seth L et al. *JAMA Network Open*.

	Final results from the PERUSE study of first-line pertuzumab plus trastuzumab plus a taxane for HER2-positive locally recurrent or metastatic breast cancer, with a multivariable approach to guide prognostication	Miles et al 2021	1
	Subcutaneous trastuzumab with pertuzumab and docetaxel in HER2-positive metastatic breast cancer: Final analysis of MetaPHER, a phase IIIb single-arm safety study	Kuemmel et al 2021	1
**Both**			
	Cardiac Tolerability of Pertuzumab Plus Trastuzumab Plus Docetaxel in Patients With HER2-Positive Metastatic Breast Cancer in CLEOPATRA: A Randomized, Double-Blind, Placebo-Controlled Phase III Study	Swain et al 2013	5
	Pertuzumab, trastuzumab, and docetaxel for Chinese patients with previously untreated HER2-positive locally recurrent or metastatic breast cancer (PUFFIN): a phase III, randomized, double-blind, placebo-controlled study	Xu et al 2020	5

© 2025 Seth L et al. *JAMA Network Open*.

eFigure 1. Funnel plots summarizing the risk of publication bias for a) T-DM1, b) T-DXd, c) TC, d) TPC.

T-DM1

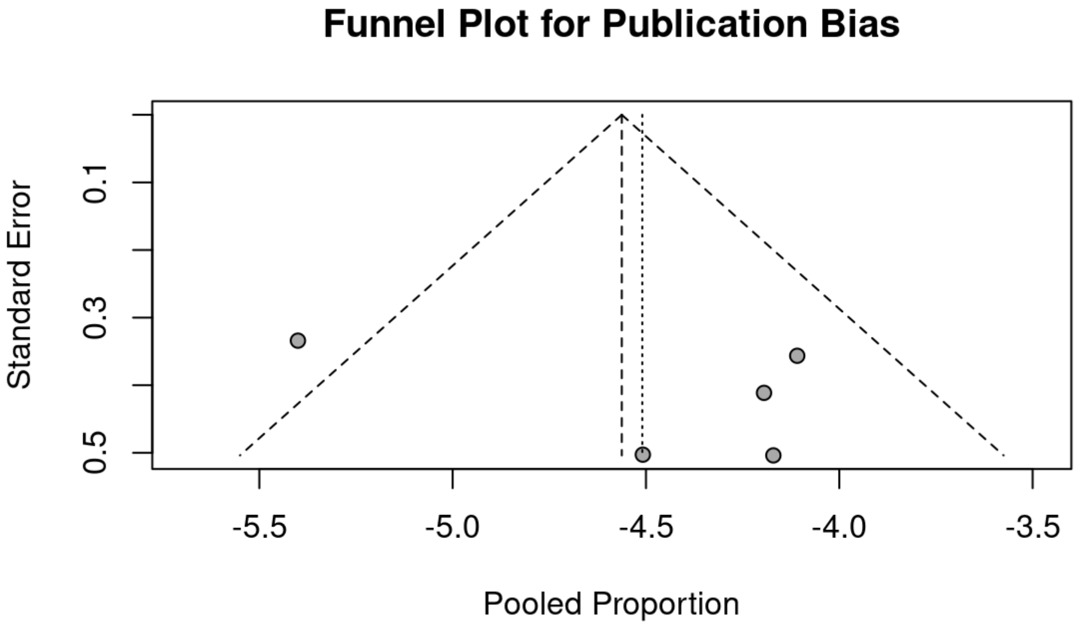

T-DXd

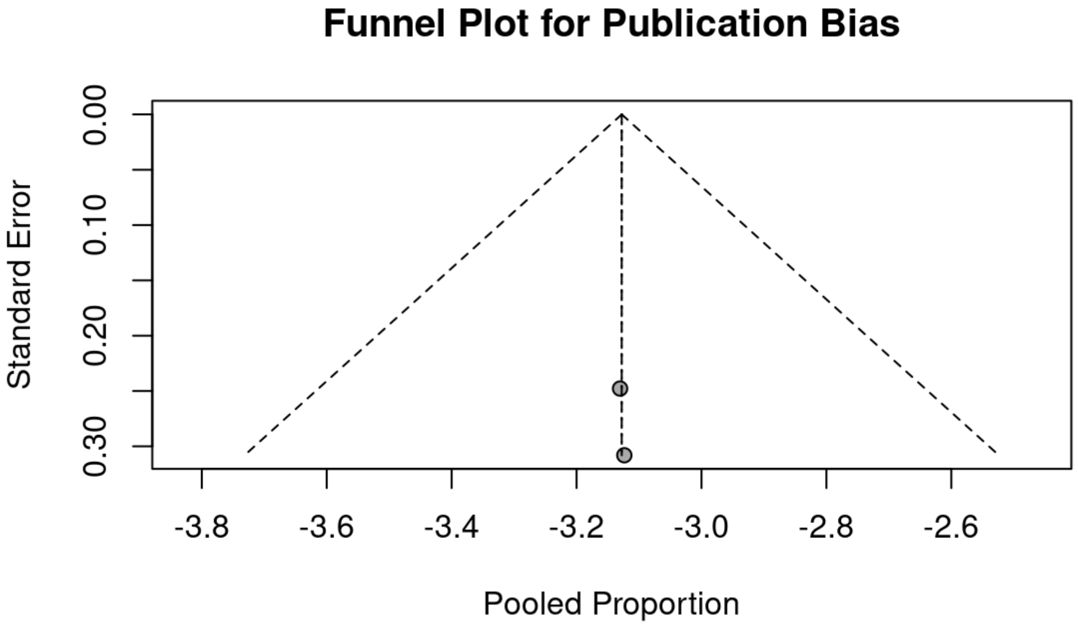

© 2025 Seth L et al. *JAMA Network Open*.

TC

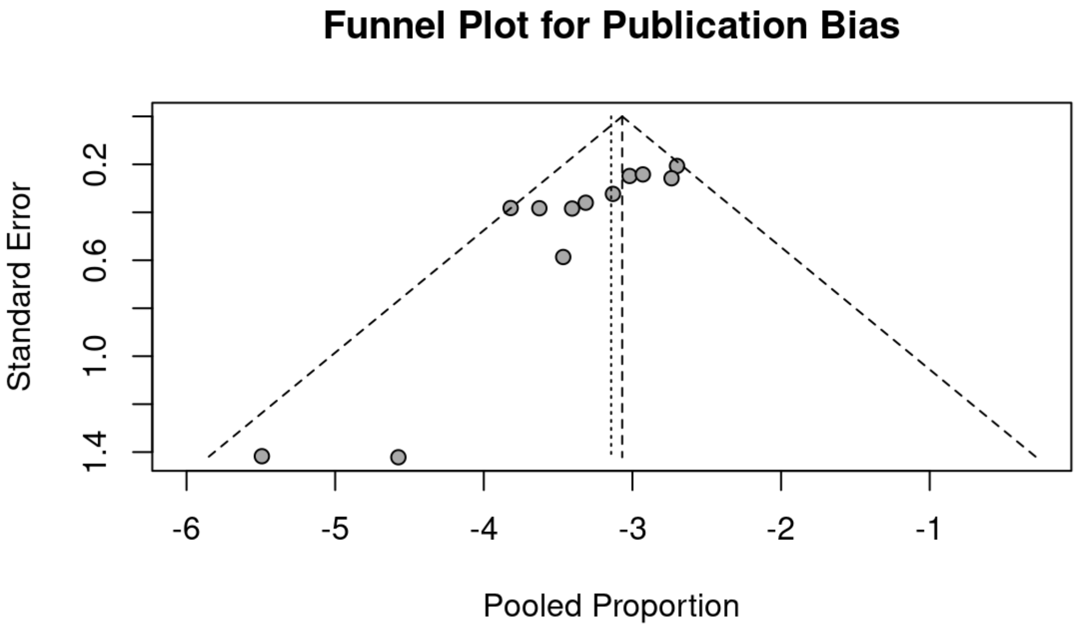

TPC

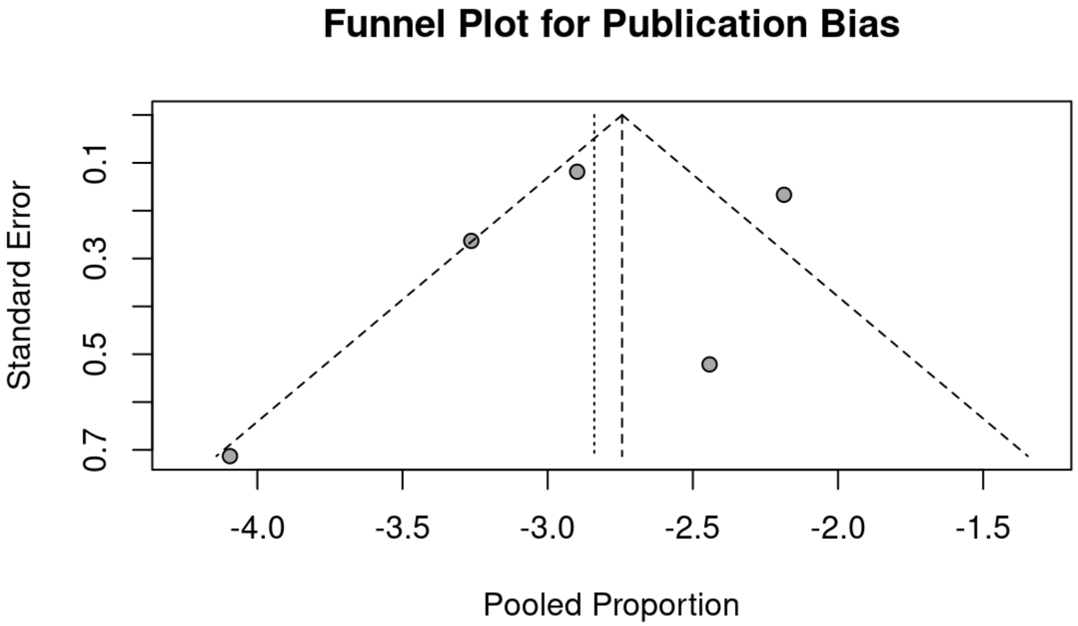

© 2025 Seth L et al. *JAMA Network Open*.

eFigure 2. Trim and fill approach adjusting for publication bias.
Shaded circles represent the original studies, and non-shaded circles represent studies that were added to adjust for publication bias for a) T-DM1 and b) TC.

T-DM1

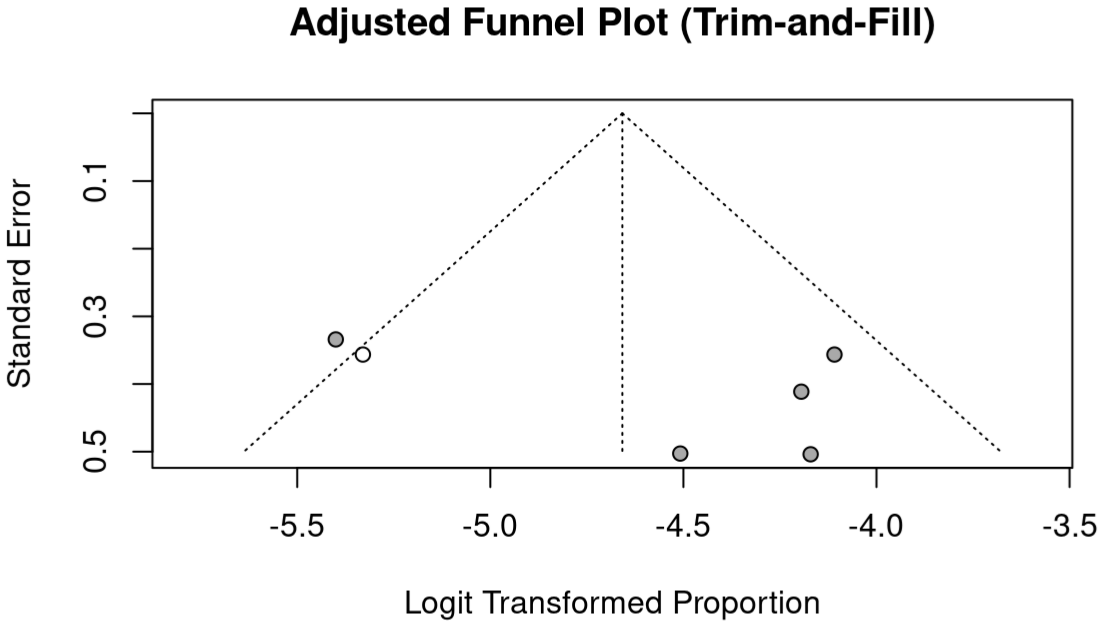

TC

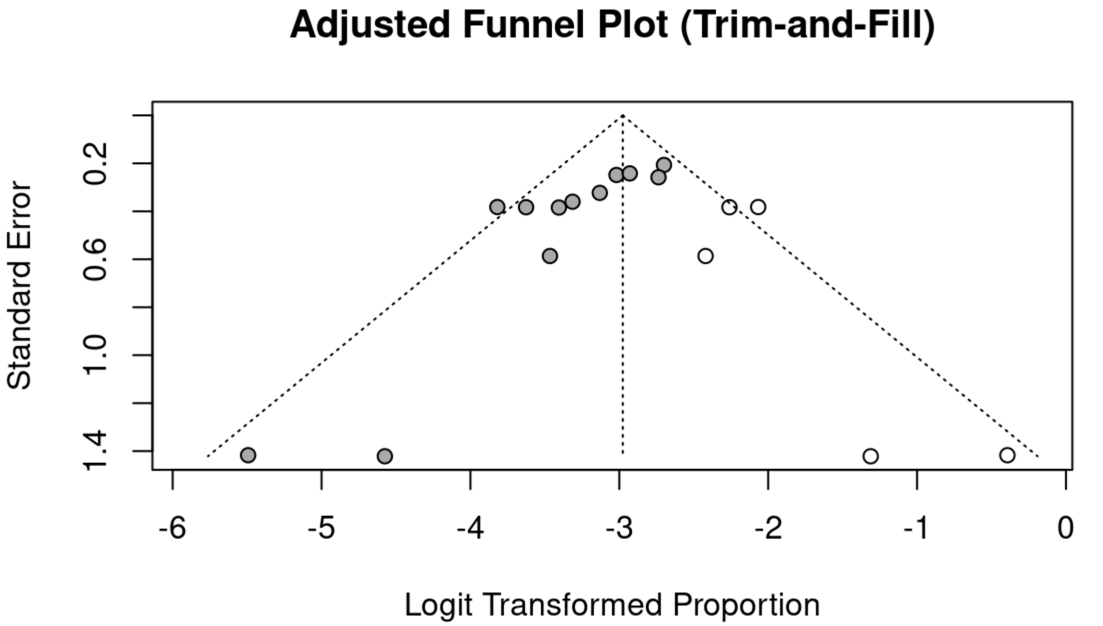

© 2025 Seth L et al. *JAMA Network Open*.
