# Supplementary material for: Cardiotoxic Effects of Antibody Drug Conjugates vs Standard Chemotherapy in ERBB2-Positive Advanced Breast Cancer: A Systematic Review and Meta-Analysis
Source: JAMA Netw Open. 2025 Nov 9;8(11):e2540336. doi: 10.1001/jamanetworkopen.2025.40336 (PMC12598513; doi:10.1001/jamanetworkopen.2025.40336)
Supplement: Supplement 2. — Data Sharing Statement [file jamanetwopen-e2540336-s002.pdf]

Data Sharing Statement

Seth. Cardiotoxic Effects of Antibody Drug Conjugates vs Standard Chemotherapy in ERBB2-Positive Advanced Breast Cancer. *JAMA Netw Open*. Published November 09, 2025. doi:10.1001/jamanetworkopen.2025.40336

Data
Data available: No
